# Supplementary figures and images for: Comparative Transcriptome and Co-Expression Network Analyses Reveal the Molecular Mechanism of Calcium-Deficiency-Triggered Tipburn in Chinese Cabbage (Brassica rapa L. ssp. Pekinensis)
Source: Plants (Basel). 2022 Dec 16;11(24):3555. doi: 10.3390/plants11243555 (PMC9785529; doi:10.3390/plants11243555)

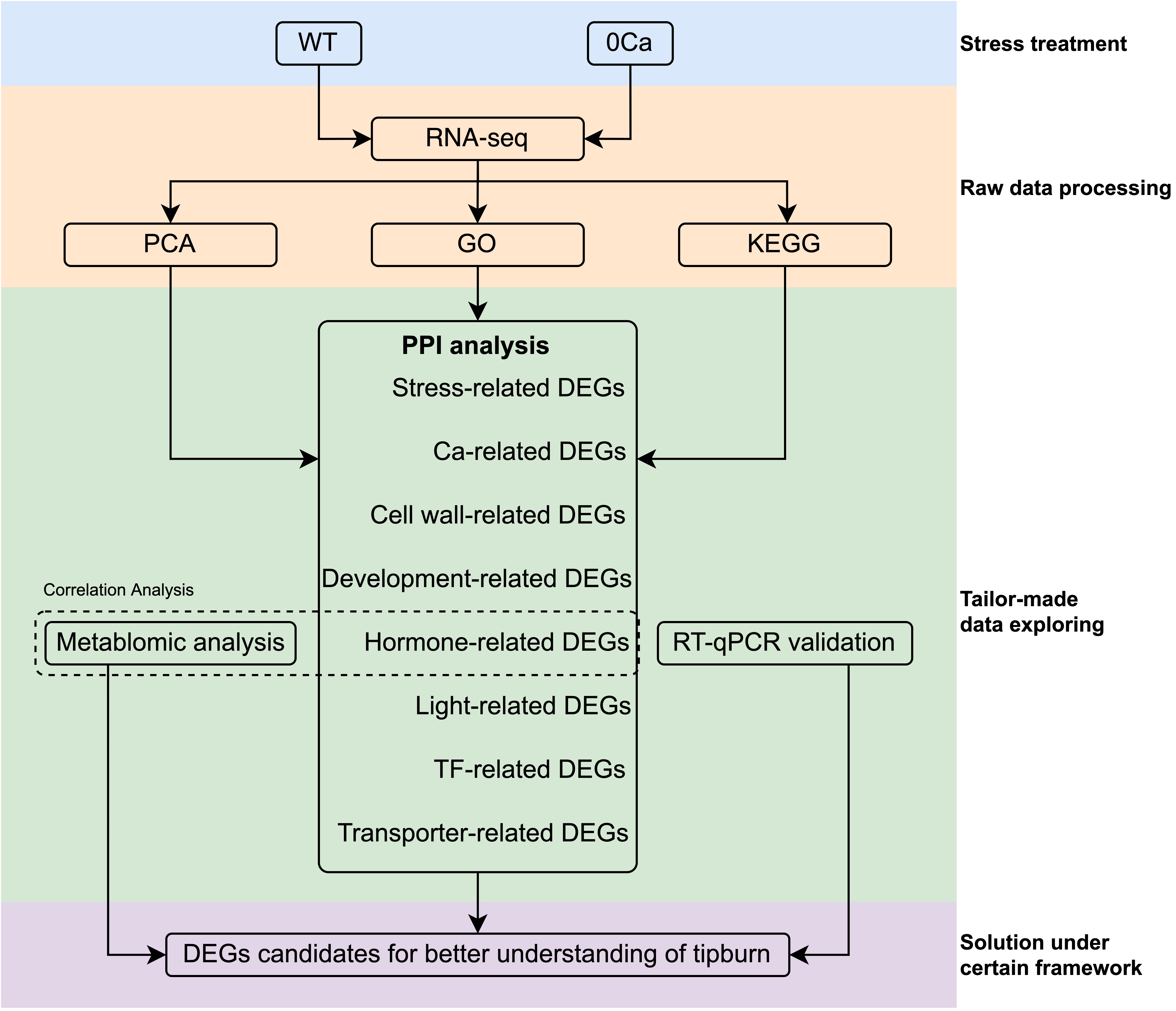

Supplement: Supplementary file 1 [file plants-11-03555-s001.zip › Figure S2 Flow Chart of experimental design.jpg]
